# Building evidence for prevention: case studies on research impacts and influences on health policy, practice, and research capacity

**DOI:** 10.1093/tbm/ibag064

**Published:** 2026-09-25

**Authors:** Lesley King, Blythe J O’Hara, Adrian E Bauman, Philayrath Phongsavan

**Affiliations:** Sydney School of Public Health, Faculty of Medicine and Health, The University of Sydney, Prevention Research Collaboration, Sydney, NSW, Australia; Charles Perkins Centre, The University of Sydney, Sydney, NSW, Australia; Sydney School of Public Health, Faculty of Medicine and Health, The University of Sydney, Prevention Research Collaboration, Sydney, NSW, Australia; Charles Perkins Centre, The University of Sydney, Sydney, NSW, Australia; Sydney School of Public Health, Faculty of Medicine and Health, The University of Sydney, Prevention Research Collaboration, Sydney, NSW, Australia; Charles Perkins Centre, The University of Sydney, Sydney, NSW, Australia; Sydney School of Public Health, Faculty of Medicine and Health, The University of Sydney, Prevention Research Collaboration, Sydney, NSW, Australia; Charles Perkins Centre, The University of Sydney, Sydney, NSW, Australia

**Keywords:** chronic disease prevention, translation, research impact, health policy

## Abstract

**Background:**

Public health research must influence both the research field and health sector. This study explored the impacts of four multi-study prevention research programs conducted by an Australian public health research group.

**Methods:**

Using a case study methodology and multiple data sources—document review, interviews, survey, and expert assessments—we studied the trajectories linking research to impact. Analyses included types of evidence generated, context, translational efforts, and impacts across four domains: knowledge development; use of research in the research system; health policy and practice; and health and social impacts. Expert reviewers assessed case studies against indicators covering each impact domain. A framework analysis was used to compare trajectories across cases.

**Results:**

Each research program was assessed as having moderate to high impact across all domains. These highly applied, policy relevant research programs achieved significant peer review publications and citations. Both policy-initiated and research-initiated programs included policy collaborators as co-authors. All case studies were policy-aligned and addressed translation factors; and the capacity of individual researchers, the research organization’s culture and the degree of collaboration were facilitators of impact.

**Conclusions:**

We found clear evidence of demonstrable policy, practice and academic impacts, with variations in impact degree and types. The differing trajectories signal the importance of assessing impacts in relation to the purpose and focus of the research. These case studies illustrate ways that research can be impactful and how classic translational enabling factors can be enhanced. Sophisticated research translation skills and a collaborative organizational culture appear key attributes for achieving impact.

## Teaser

This study examines how four Australian prevention research programs achieved real-world impact across knowledge, policy, practice, and research capacity. It shows that with the pathway from research to impact, close collaboration with policy partners and strong translation skills are consistently essential for success.

## Graphical Abstract

**Graphical Abstract:**
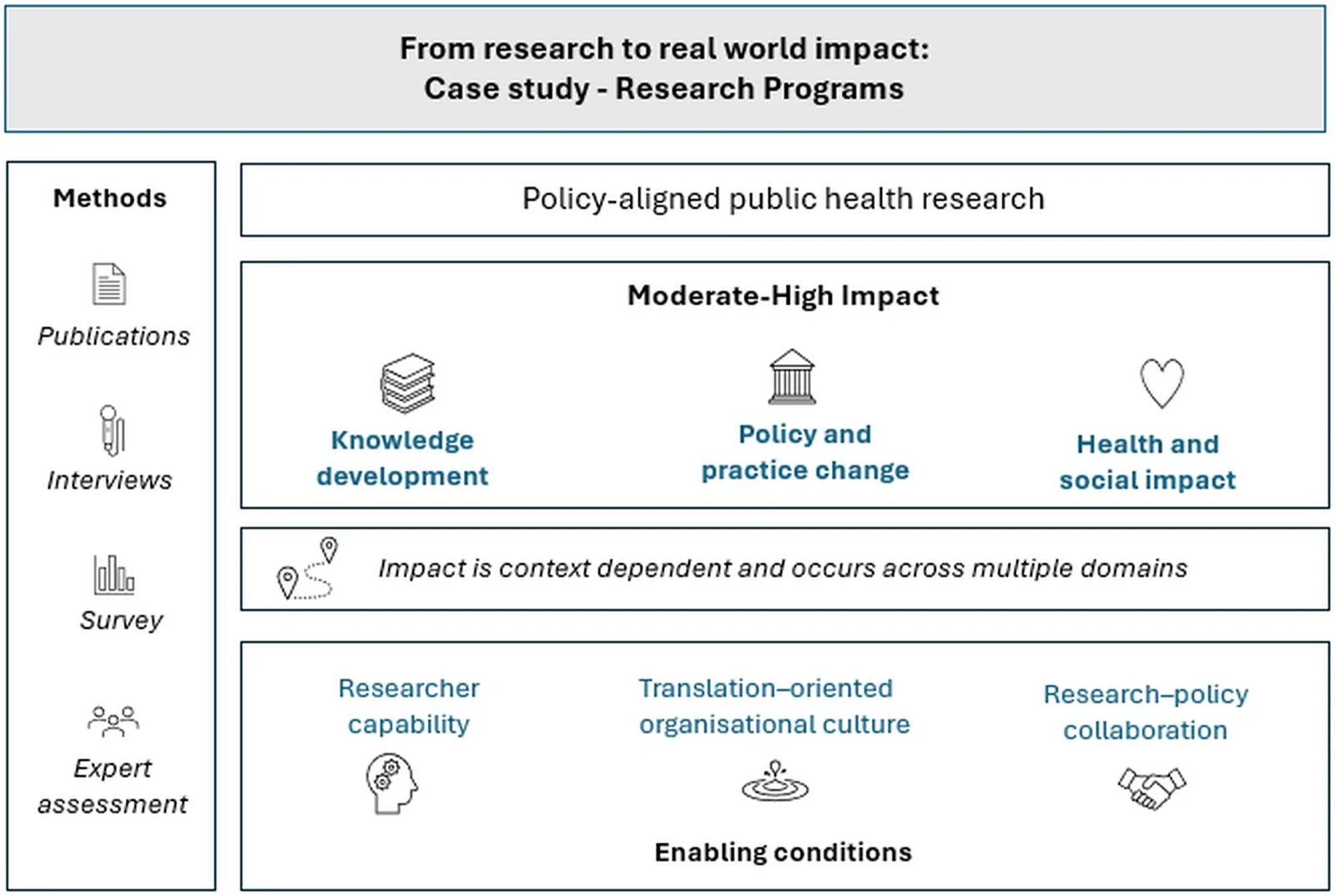

Implications **Practice**: Achieving public health research impact requires researchers to develop translation skills and invest in sustained, trust-based relationships with policy and practice partners, recognizing that the pathway from evidence to impact will differ depending on the nature and purpose of the research program.

**Policy:** Policymakers should pursue co-production models in which practitioners are embedded as genuine collaborators in research programs from the outset, while also broadening how they define and measure the value of public health research investment so as to move beyond publication outputs to capturing contributions to policy development and practice change.

**Research:** Future research should move beyond case study descriptions to develop and test frameworks that can prospectively evaluate how organizational culture, researcher translation capacity, and the depth of policy collaboration each contribute to research impact across different types of prevention research programs.

## Introduction

Understanding the impact of public health-related research on both the research field and the health sector has long been the subject of academic enquiry [1, 2]. In recent years, this has been accompanied by increasing demands from funders, policymakers and academic institutions to demonstrate relevance and impacts [3]. From a policy and program perspective, there have been growing expectations for evidence-based decision-making in health, along with increasing clarity about the value of different types of research evidence. Types of evidence in public health can be viewed as a set of building blocks, encompassing problem definition, solution generation, innovative testing/intervention demonstration, intervention translation, and scaling up [4, 5]. Such frameworks can be used to profile the research conducted across an organization or within a research program and serve as a basis for analyzing the range and types of impacts of a given research program or organization.

Several approaches have been developed to determine the impacts of research. The most popular being the Payback framework [6–8], which was first developed in response to demands to justify public expenditure on health research. The framework has been refined over time and applied across a number of health research contexts [7]. The Payback framework has proven valuable, identifying multidimensional impacts and drawing on multiple data sources [8]. It has also generated various adaptations for varied contexts and settings, including that of Cohen *et al.* [9]and Ward *et al.* [10]; both of which retain the Payback framework’s emphasis on multidimensional impact categorization and multiple data sources, including insights from end users [7, 11], while adapting the specific domains and data collection processes for application to complex research programs and organizations, and public health-specific translational contexts. With increased expectations from academic institutions and peak research funding bodies, frameworks have increasingly addressed impact pathways within the academic domain [10].

At the same time, a more inclusive understanding of the complex mix of factors underpinning policy and practice [12, 13] has led to a broader conceptualization of impact which includes “research influence,” and not just direct adoption of interventions [10]. Given the fluidity of policy priorities and political considerations, alignment of research with policy can be taken as a measure of policy/practice relevance, and meaningful even if not subsequently impactful [10]. The concept of “influence” can thus include policy alignment, generation of evidence related to given policies or use of findings in relation to other evidence. Similarly, a clearer distillation of policy processes also fosters caution about drawing links between single research studies and distal impacts, such as health and social changes [9, 10]. It is widely recognized that the conditions in which impacts occur include factors beyond the research itself, including features of the research and policy environments [12].

Despite differences in emphasis, there remains strong consistency in the frameworks presented in the literature regarding the broad domains of impact [7, 9, 10, 12, 14]. They typically cover: knowledge development; building research capacity, skills and experience; the use of research in informing policy and practice decision making, and in an indicative way, health and social/societal impacts.

Any impact assessment requires a balance between accuracy, comprehensiveness and feasibility, and recognition that impacts may vary over time. While there is ongoing interest in routine monitoring approaches to assessing impacts, research studies have typically undertaken more detailed investigations, drawing on multiple sources of data, and combining case study narratives with relevant qualitative and quantitative indicators [9, 10, 15, 16]. However, few studies have applied these frameworks at the level of multi-study research programs (that is sustained bodies of inter-related work) within specific policy context, such as Australian public health [12].

Drawing on the accumulated experience in impact assessment, this study explored the impact and influence of selected research programs conducted by an Australian research group specializing in chronic disease prevention. A research- and postgraduate teaching-intensive group, the Prevention Research Collaboration (PRC) is based at the University of Sydney and has a 20-year history of undertaking policy-relevant research and engaging with policymakers and practitioners to support evidence-based policy and practice. PRC also has a significant and sustained track record in conducting a mix of policy-initiated and investigator-initiated research, both generating various types of evidence from descriptive to evaluative [17]. While the recent descriptive account of PRC research indicates good alignment with Australian public health policy priorities [17], specific impacts have not been systematically documented, beyond case studies of child obesity research [13, 18]. This article investigates the broad impacts of four research programs on health-related policy and practice and public health research capacity, as well as the conditions and contexts in which these programs were implemented, and how these may have influenced translational processes and impacts. Of interest are potential differences in the trajectories and patterns of policy-initiated and investigator-initiated research programs.

## Methods

### Study design

A case study methodology (using a mixed method approach) examining substantial research programs was adopted to allow in-depth analysis of the trajectory and impacts of each program [19, 20]. Case study methodology enables comprehensive examination of complex phenomena within their real-world contexts, making it particularly suitable for understanding trajectories of how research translates into policy and practice outcomes. Research programs, comprising multiple inter-related studies and outputs rather than single projects or studies, were selected as the unit of analysis, as the literature indicates that they can build a body of complementary evidence that is potentially sufficient to underpin real-world changes and credibly add to the knowledge base in a given topic [12, 21].

A mixed-methods approach [22] was adopted because no single data source could adequately capture the breadth of impact domains under investigation: document review provided evidence of outputs and reach, interviews and surveys captured stakeholder perspectives on processes and less tangible impacts, and expert reviewer ratings enabled structured, comparable judgements of impact magnitude across case studies. Integrating these sources allowed for triangulation and a more complete account of impact than any single method could provide. This design combined sequential and concurrent elements. Document review, including bibliometric analysis, was conducted first, informing the development of the interview guide (sequential exploratory phase). Key-informant interviews and the online survey were then administered concurrently for each case study (concurrent phase). Expert reviewer assessment was subsequently undertaken, drawing on the case study documentation and summarized findings from the preceding phases to inform reviewers’ ratings and free-text commentary (sequential phase). All data sources were integrated during analysis and reporting. Qualitative data were prioritized for explanatory depth, with quantitative data (bibliometric indicators, reviewer ratings) providing corroborating and comparative evidence.

### Case study selection

Four case studies were purposively selected from research programs conducted within the PRC. Programs were eligible for inclusion if they: (i) comprised a substantial body of inter-related studies and outputs, rather than a single project, consistent with our unit of analysis; (ii) were either completed or had progressed sufficiently to allow meaningful assessment of impact at the time of case selection; (iii) had generated sufficient documentation (publications, reports, correspondence) and identifiable stakeholders to support multi-source data collection; and (iv) collectively represented variation in program origin (policy-initiated vs. investigator-initiated) and topic area, to allow exploration of how these features may influence trajectories. Further, each program involved different research teams (that is with different mix of researchers) within the PRC. Applying these criteria, four case studies were selected. Two case studies were policy-initiated research programs, namely Evaluation of the get healthy service research program (herein referred to as GHS) and evaluation of the healthy facilities research program (herein referred to as HSF); and two were investigator-initiated, namely sitting, sedentary behaviors and health research program (herein referred to as Sitting) and generation vape research program (herein referred to as GenVape) (Table 1).

**Table 1:** Comparative overview of the four research programs and their implementation.

	Get healthy service evaluation (GHS)	Healthy NSW health facilities evaluation (HSF)	Generation Vape research (GenVape)	Sitting and sedentary behaviors research (Sitting)
Description	The GHS research program covered the evaluation of the implementation of a free population-wide telephone/web obesity prevention, behavior change program, covering process and impact evaluation related to recruitment, retention and outcomes.	This program evaluated the implementation of healthier food policies in NSW Health services’ facilities. Studies generated evidence on the feasibility, acceptability and impacts of this policy.	GenVape is a research collaboration examining the vaping attitudes, beliefs, and behaviors of parents, teachers, and Australians aged 14-24 years (both users and non-users). It tracks young people’s vaping and smoking behaviors and impact of policy, programs, and education initiatives.	The Sitting research program was predominantly a series of epidemiological studies exploring the role of sitting as an independent risk factor for chronic disease. Other strands comprised intervention development and testing, conducted in collaboration with the NSW Heart Foundation and other disciplines.
	Funded by NSW Health and commenced at the same time as the service.	Funded by the NSW Government and commenced at the same time as the policy.	Funded through research grants and non-government organizations.	Funded through research grants for sitting research.
Approximate timeframe of research program	2009–15	2017–22	2022–25	2010–14
Initiator	Policy-initiated	Policy-initiated	Investigator-initiated	Investigator-initiated
Partnership/co-design	Government—from inception	Government—from inception	Non-government collaborator; subsequent collaboration with government	Non-government collaborator for solution development and testing
Focus	Intervention process and impact evaluation with rapid translation into service implementation.	Intervention evaluation with rapid translation into program implementation.	Problem analysis with a focus on influencing policy action.	Epidemiological studies focusing on problem analysis and subsequent solution testing.
Topic	Intervention was pre-determined; dependent on a window of opportunity and funding.	Intervention was pre-determined; dependent on a window of opportunity and funding.	Vaping was an emerging health risk with limited evidence.	Sitting and sedentary time was emerging as aa potential risk factor.
Scale	Population-wide level for state jurisdiction (later scaled to other jurisdictions across Australia).	Across all state health services/organizations.	National population-wide level (started initially in one jurisdiction).	Epidemiological studies used state, national and international datasets. Intervention studies were local, in selected workplaces.
Setting	Telephone coaching	Food retail environment	Media advocacy; legislation.	Workplaces; professional settings
Target group	Adults with chronic disease risk factors	Health service staff and visitors	Young adults; adolescents; legislators	Adults; environments
Number of qualitative data sources	*N* = 2 Researcher interviews	*N* = 3 Researcher interviews	*N* = 1 Researcher interview	*N* = 2 Researcher interviews
	*N* = 2 Policy interviews	*N* = 2 Policy interviews	*N* = 2 Policy interviews	*N* = 2 Policy interviews
	*N* = 5 survey respondents	*N* = 0 surveys	*N* = 5 survey respondents	*N* = 5 survey respondents

Note: NSW Health is part of the New South Wales state government, responsible for strategic policy directions and operations of public health services in NSW, the most populous state in Australia.

### Conceptual framework

For each case study, we collected information across four domains reflecting our adapted framework for understanding research impact: knowledge development; policy and practice changes; research capacity and environment; and social and health outcomes. Additionally, information was collected about the policy and research contexts in which the work was undertaken and the researchers’ translational efforts to apply their findings (Fig. 1).

**Figure 1:**
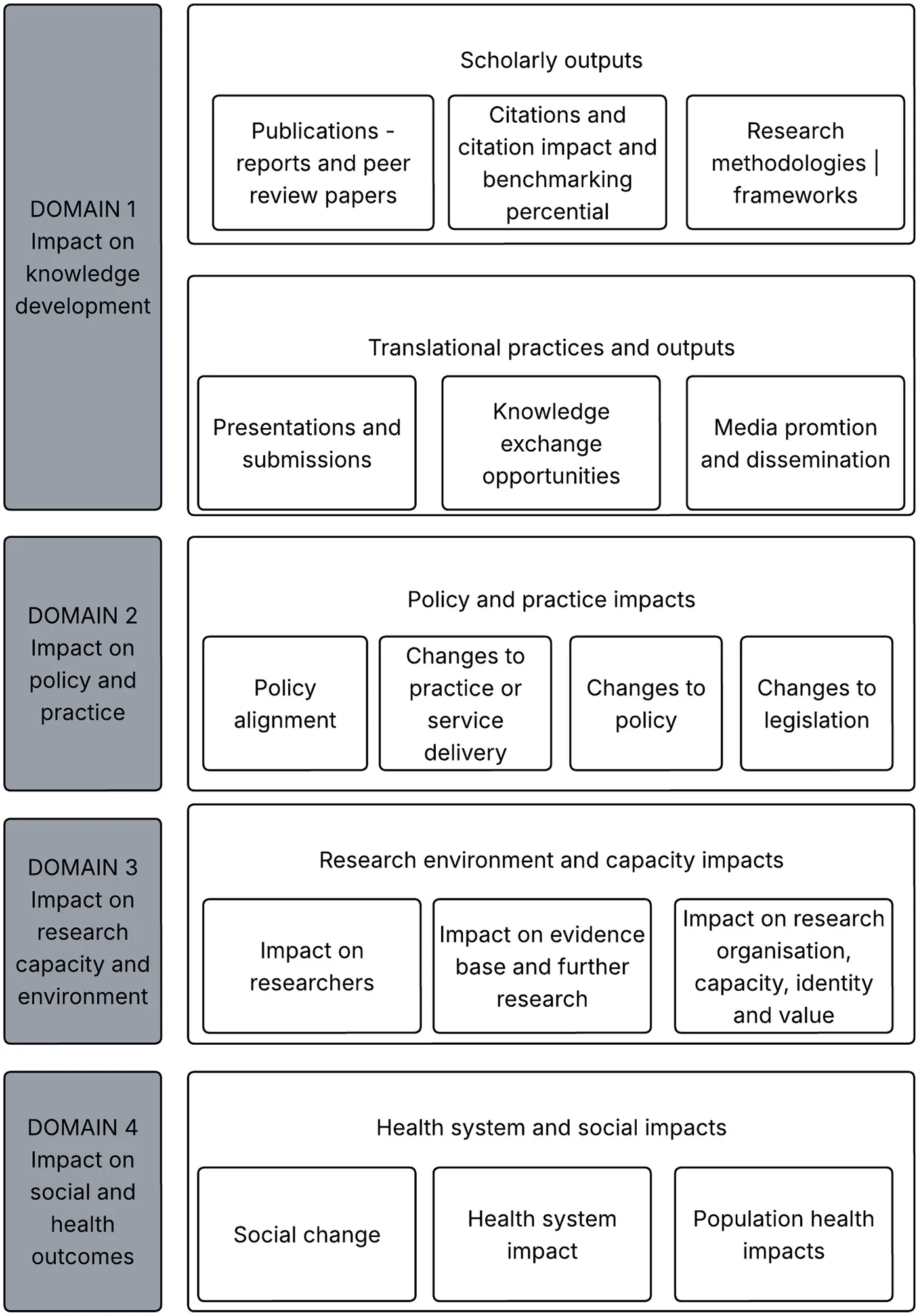
Impact domains (adapted from Cohen et al. [9] and Ward et al. [10]).

### Data collection

Information was collected from multiple sources in line with previous research [7, 9, 10, 12, 14, 19, 23]*:* document/publication review; key informant semi-structured interviews; online survey; and expert reviewer assessments. The study was approved by the Sydney University Ethics committee (2024/HE000266). Data collection occurred over a 6-month period. Each case study was undertaken by a single researcher (and author). All participants provided consent.

Interviews for each case study were conducted by an author (B.O.H. or L.K.) not directly involved in delivering or evaluating that program, to reduce bias. Three interviewees (B.O.H., P.P., and A.B.) were also co-authors of this manuscript but were not involved in conducting or analyzing data for the case study in which they were involved. The interviewers and interviewees are based within the same broader institution (PRC), which may have influenced interviewee rapport and disclosure. These relationships and their possible influence were discussed by the research team throughout data collection and analysis.

An overview of methods is provided in Fig. 2.

**Figure 2:**
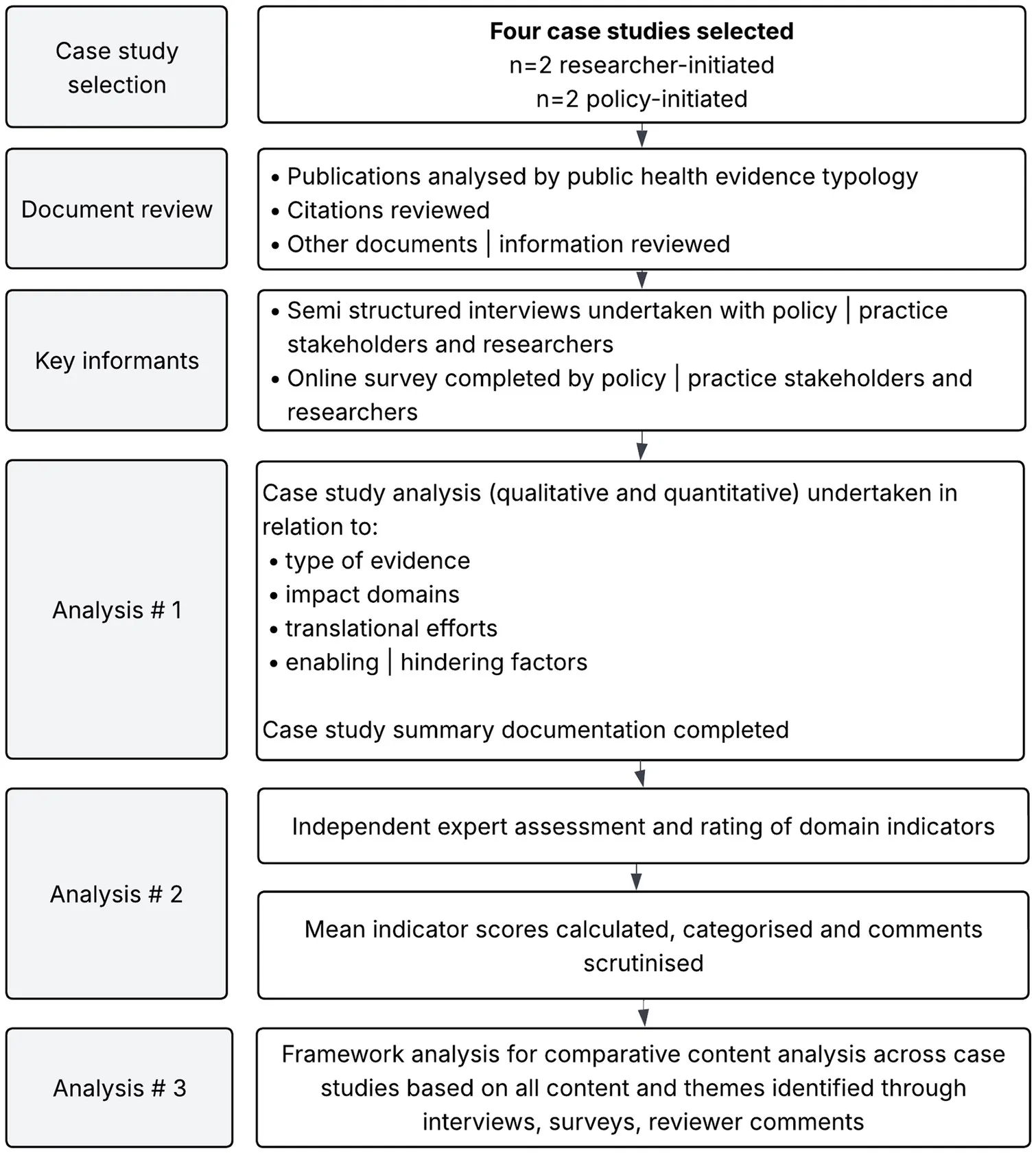
Overview of methods.

#### Document/publication review

A comprehensive document review was conducted covering peer-reviewed publications, policy documents, reports, presentations, and media releases and, where relevant to specific case studies, parliamentary records (e.g. Hansard transcripts and parliamentary and senate inquiry reports). Documents were identified through a combination of reviewing PRC publication listing and internal reports and supplemented by materials nominated by key informants during interviews. The review documented research outputs, translational activities, and evidence of impact across the four domains. This information was summarized along with a bibliometric analysis examining the total number of citations for all peer review publications arising from each project [19].

#### Key informant semi-structured interviews

Interviews (approximately 30–60 minutes in duration) were conducted with researchers, policymakers, and practitioners relevant to the case study. Interviewees were purposively sampled to represent key roles (research leads, policymakers, and practitioners) within each program, supplemented by snowball sampling via participant nomination [24]. Initially interviewees were identified by the authors based on their authorship of publications and known links to policy and practice actors/agencies. A total of 16 interviews and 15 surveys were completed across the four case studies (interviews’ range: 3–5 per case; surveys’ range: 0–5 per case, with no survey responses obtained for the HSF case study). The interview guide was developed by the research team (B.O.H. and L.K.) based on the four impact domains of the conceptual framework (Fig. 1) then piloted and refined prior to use. The interviews covered participants’ role and work context and scope of the research program under investigation. They also explored policy and program impacts resulting from the research, contributions to knowledge development in the field, processes implemented for translating this research into practice, and implications and impacts of the research work on the personal and organizational capacity of known stakeholders. Interviewees were also asked to nominate additional participants to take part in an interview or undertake an online survey.

Each key informant was contacted by email and provided with participant information and consent forms. All interviews were transcribed using MS Teams and responses deidentified.

#### Online survey

An online survey of researchers and policymakers was developed and accessed via the Qualtrics platform and covered the same topics as the key-informant interviews. Surveys were provided to those who were not available for interview or were more peripherally involved in the research program.

#### Expert reviewer assessment

An expert review process was implemented using four independent reviewers with subject-matter expertise in research and policy implementation within public health (two senior research academics; two highly experienced policymakers/practitioners). They were selected to ensure no conflict of interests. Reviewers were not required to have specific expertise in case study or mixed-methods research methodology; their role was to assess the evidence and credibility of research program impacts, as documented in the study, not to evaluate the study design. Each reviewer was assigned two case studies (see data analysis section on case study reports analysis) and provided with documentation, impact summaries, and standardized indicator rating tools. Reviewers independently provided numerical ratings (1–5 scale) and explanatory comments, justifying their assessments based on evidence presented and their expert judgment. Researchers provided brief commentary for each indicator, to aid reviewer deliberations, without revealing their own ratings (assessment materials can be provided upon request).

### Data analysis

As outlined below, data analysis involved integration of qualitative and quantitative data at multiple points: first, for one of the two main researchers to prepare a case study report for each program; second to incorporate expert reviewer ratings for each program; and third to facilitate a comparison of impacts and trajectories across case studies. All coding data were discussed to ensure consistency and reduce interpretative bias. Saturation of content/themes was not possible given the small number of programs covered and limited key informants for each case study.

A single (experienced) researcher (B.O.H. or L.K.) undertook document review and interview analyses for each research program. A researcher (B.O.H.) experienced in both quantitative and qualitative data analysis undertook the online survey analysis. Our coding approach was primarily deductive, with interview material and free text online survey material initially coded according to pre-determined domains, as prior exploratory research had identified these established impact categories. Coding decisions were discussed with a second team member to sense-check interpretations and support consistency across case studies. This structured approach is consistent with that described by Rendle *et al.* [25] and Ramanadhan *et al.* [26]. The coding involved integrating qualitative and quantitative data (document review findings, interview data, and survey data) by impact domain, in order to compile an integrated case narrative for each program (see *case study reports*, below).

Second, quantitative expert reviewer ratings were integrated with their reviewer commentary to resolve scoring discrepancies (see *impact assessment process and rating*, below). Third, qualitative data from interviews, surveys, and reviewer comments were compared across all four case studies to identify cross-cutting contextual themes (see *framework analysis*, below).

### Case study reports

Case study reports presented integrated accounts summarized under key headings aligned with our impact domains. These case study reports included substantial excerpts from transcribed interviews and survey comments, and summaries of domain impacts. This approach was consistent with items identified in the research impact assessment reporting checklist [11].

#### Impact assessment process and expert reviewer rating

Drawing on impact assessment approaches and indicators used in previous studies [10, 27], we developed a set of impact assessment indicators covering the four impact domains of the conceptual framework, and translational efforts. A 5-point scale (1 = None, 2 = Minimal, 3 = Moderate, 4 = Major/High, 5 = Substantial/Very High) was used to rate each indicator (Table 2).

**Table 2:** Average impact assessment scores for each case study.

	Get healthy service evaluation	Healthy facilities evaluation	Generation Vape research	Sitting and sedentary behaviors
Impact domain: knowledge development	Major impact	Moderate impact	Major impact	Substantial impact
New knowledge/evidence added	Substantial impact	Major impact	Substantial impact	Substantial impact
Follow-on research fostered	Major impact	Moderate impact	Major impact	Substantial impact
Citation levels	Major impact	Moderate impact	Major impact	Substantial impact
Translational processes	Moderate impact	Moderate impact	Substantial impact	Major impact
Media coverage	No impact	Minimal impact	Substantial impact	Substantial impact
Stakeholder engagement	Major impact	Moderate impact	Substantial impact	Major impact
Translation factors considered	Major impact	Major impact	Substantial impact	Major impact
Impact domain: policy and practice	Major impact	Major impact	Substantial impact	Major impact
Alignment with global priorities	Major impact	Major impact	Moderate impact	Major impact
Public health significance	Major impact	Moderate impact	Substantial impact	Major impact
Alignment with Australian/NSW priorities	Major impact	Major impact	Substantial impact	Major impact
Impacts on policy	Major impact	Moderate impact	Substantial impact	Substantial impact
Impacts on practice	Major impact	Moderate impact	Major impact	Major impact
Reach of policy/practice impacts	Moderate impact	Moderate impact	Substantial impact	Substantial impact
Impact domain: research capacity and impact	Major impact	Moderate impact	Major impact	Substantial impact
Skills/career development	Major impact	Moderate impact	Major impact	Major impact
Organization identity/value	Substantial impact	Moderate impact	Substantial impact	Substantial impact
Impact domain: social and health impact	Major impact	Moderate impact	Major impact	Major impact
Social change impact	Moderate impact	Moderate impact	Major impact	Major impact
Health system impact	Major impact	Moderate impact	Major impact	Major impact
Population health impact	Moderate impact	Minimal impact	Major impact	Major impact

Based on the expert reviewer assessment of indicators, the mean reviewer scores were calculated and categorized. This design ensured each case study received assessment from two independent expert reviewers. Authors (B.O.H. and L.K.) separately completed joint impact ratings, and all scores and comments were systematically collated within case studies. Where there were notable discrepancies between reviewers’ scores (that is, cases where there were category differences in reviewers’ impact scores: for example, moderate versus high impact), reviewers’ comments were scrutinized to understand the rationale for each score. We found that the source of these differences was explained by either focus or timing factors: the focus was on a different impact (e.g. policy or behavioral), or a potential rather than actual impact; or the timing of when alignment with government policy (initial or later stages) was considered.

### Framework analysis

Whereas the case study reports (above) integrated data sources within each case, framework analysis was used to compare qualitative and quantitative content across cases. In qualitative research, comparing content across cases is known as framework method analysis [28]. Key content and themes identified across data sources were extracted for each case study and tabulated. In addition, further examination of interview transcripts, survey responses and reviewer comments were undertaken, to uncover any additional themes and details related to the context and circumstances that may have enabled or hindered the attainment of impacts, including translational processes. This more inductive method of coding of enabling factors sought to identify more fine-grained exploration of issues associated with the degree of impact for each case study.

## Results

### Research program approaches

An overview of the four research programs is presented in Table 1. Two cases were policy-initiated, with predetermined topics and government partnerships from inception (GHS; HSF). The policy-initiated research programs were both State-level intervention evaluations, whereas the investigator-initiated programs (GenVape, Sitting) were each focused on problem identification and analysis, and subsequently used findings to support interventions, and in the case of Sitting, to investigate interventions. All research programs were conducted on a large scale, involving analyses of large datasets, population-level surveys, or the evaluation of at-scale implementation (across a whole state). Citations gave an indication of international relevance in all cases.

### Impact assessment ratings

The average impact assessment scores across the four case studies suggest that all research programs made meaningful translational efforts and had a moderate to high impact across the four impact domains (Table 2).

### Knowledge development impact

As multi-study research programs, all cases contributed to building the evidence-base within their topic area. The profile of types of evidence produced in each case is shown in Table 3.

**Table 3:** Number of publications by study type for each case study.

	Problem description	Methodology development	Solution generation	Intervention evaluation	**Combined citations and average field weighted citation impact (FWCI)** ^a^ **(range)** *As at February 2025*
Get healthy service evaluation (2011–19)		3		15	18 papers 456 citations Average FWCI 1.93 (1.60–2.97).
Healthy NSW health facilities evaluation (2020–23)			2	3	5 papers 42 citations Average FWCI 0.97 (0.43–1.62).
Sitting and sedentary behaviors research (2010–22)	21	5	6	11	43 papers—*citations below based on analysis of 5 publications* 1427 citations Average FWCI 5.28 (4.01–8.53)
Generation Vape research (2022–24)	5				5 papers 120 citations Average FWCI 3.91 (1.17–7.97)

a FWCI shows how well cited a publication is when compared to similar publications. A value greater than 1.0 means the document is more cited than expected according to the average. It takes into account: the year of publication, document type and disciplines associated with its source [29].

Three of the four cases were scored by expert reviewers as having high impact on knowledge development; with the HSF case scored as having moderate impact in this domain. All cases produced peer review publications, although the number of publications varied considerably (Table 3). Citation figures show they all received some (in some cases very high) international interest: there was a high degree of citations for the researcher-led Sitting and GenVape work; substantial citations for some GHS publications; and a smaller response for HSF papers. All cases involved co-design of research and co-publication with policy/practice stakeholders.

In terms of knowledge development, one expert reviewer noted the GHS research represented a “rare, comprehensive, long-term program of research that has produced comprehensive range of original research outputs across a spectrum of outcomes” (expert reviewer herein referred to as expert), providing “a translational research model” (expert). The HSF program “made proportionately higher contribution to new knowledge in this field” due to “the low existing volume of research of this type on this issue” (expert). This research addressed fundamental questions about the implementation and effectiveness of healthy facility initiatives that had previously received limited scholarly attention. GenVape was explicitly initiated “to address a lack of concrete data on the extent and nature of the use of e-cigarettes by young people” (policy key informant herein referred to as policy) and it provided “new, ground breaking research” (expert). The Sitting program “broke new ground, identifying and later refining the significance of sitting as a chronic disease risk factor” (expert).

Each research program demonstrated notable methodological innovations that enhanced their research contributions. For example, a GHS publication formalized the key step of ‘formative translational research’ [30]. The HSF program involved a “detailed and original methodology, tracking implementation and commercial indicators” (expert); and GenVape refined and adapted its research methods over time, employing a mixed methods design with an investigation of “how you ask the questions, to make sure that young people are answering them right” (research key informant herein referred to as researcher).

Furthermore, the research programs were responsive to emerging trends and policy needs. For example, GenVape “investigated under-explored aspects of vaping and tobacco/nicotine use by young people” (policy). Sitting also responded to policy interests: “PRC was responsive to the need that the Heart Foundation had to actually try and gather some insight or some evidence around sit stands” (researcher).

All case studies emphasized a deliberate and active approach to dissemination and translation. All incorporated studies that took account of translation factors, with scores on this indicator noting a high impact. They also actively disseminated research findings to reach relevant audiences, often prioritizing reporting on findings over traditional academic cycles of journal publications. Nevertheless, academic publications were important for translation: publications were described as “adding credibility to the research area and the ongoing work” (policy).

The two researcher-initiated programs demonstrated approaches to dissemination that maximized their reach and impact beyond traditional academic channels. In the case of GenVape, media advocacy was dominant, reflecting a strong sense of purpose to achieve legislative action. The research team delivered 27 presentations, launched a quarterly newsletter, and developed various promotional resources (infographics, videos). Their advocacy-focused dissemination approach prioritized timeliness and public engagement. As one policy representative explained, “you need a new study all the time to back up what you’re advocating for.” One expert reviewer noted that “the media strategy has successfully translated research into public discourse, influencing norms, awareness and advocacy efforts, increasing allies and amplifying impact.”

The Sitting program also generated widespread media interest. In response to overzealous media hype, researchers “were trying to have a more nuanced discussion or debate about this new behavior” (researcher). There was also demand for practical guidance and the Heart Foundation actively disseminated the “sit less” approach and progressively incorporated it into their policies and guidelines. As one policy representative observed, “we were getting a lot of pressure from other workplaces to give guidance …. people wanted to know how much sitting less, how often.”

By contrast, the central translational strategy of the two policy-initiated intervention evaluation cases was to rapidly communicate findings directly to policy/implementation stakeholders and collaborate on interpretation. This was deemed critical for these policy embedded evaluations.

### Impacts on policy and practice

All four research programs were scored as being aligned with global, Australian and/or NSW Health policy, with scores in high or moderate categories. Sitting and GenVape informants explained that these topics were initially aligned to broader priorities (physical activity, smoking), but that their findings subsequently led to policy adjustments.

All cases had solid evidence of actual, demonstrable policy and/or practice impacts; but the types of impacts were distinctly different. The GHS had direct operational impact, with ongoing investment, sustained implementation and adaptations for diverse target populations. It also provided a new service delivery medium for NSW health services. The HSF impact included environmental changes in relation to modifications to the availability, formulation, and portion sizes of food and beverages available in health facilities, which subsequently influenced consumer purchasing and consumption patterns. GenVape research contributed evidence supporting national legislation that banned non-pharmacy vape sales, corroborated by the document review [31, 32] and stakeholder-reported accounts of the program’s role in the policy process. The research also informed curriculum development and educational resources for schools and parents. Additionally, Sitting research led to the incorporation of sitting as a recognized risk factor in health policy and guidelines, and workplace environmental modifications including the adoption of sit-stand desks and influencing commercial availability.

The direct and immediate impacts of the two intervention evaluations were on implementation and demonstration of intervention effectiveness sufficient for service continuation and extension. Research findings were used to continuously improve their program. As one researcher explained, “the information that was being produced was used to change things to make sure that the GHS reaching those who it was meant to reach” (researcher); and further, the “[Government] continued to invest in the service and added new modules,” diversifying the service to address new priorities and reach difference target audiences. This was endorsed in expert review: “good evidence of continuous learning across the life of the program.”

For HSF, the process evaluation was used to overcome implementation challenges. A policy representative noted: “The findings from the research were used to address specific concerns and objections from Local Health Districts [local health regional units] and retailers,” for example demonstrating that “the removal of sugary drinks did not lead to significant displacement.” The findings for HSF and GHS provided evidence to implementation and funding bodies that the service or policy worked. For example, the findings confirmed that the HSF intervention “restricted the availability of unhealthy foods across NSW health facilities and changed staff and visitors purchasing behaviors” and “resulted in changes in commercial practices (expert).” For the GHS, the research findings underpinned ongoing investment and sustained adoption, “the body of evidence that we accumulated over time really helped to reassure the [Government] that they’ve invested appropriately” (researcher).

The investigator-initiated cases, GenVape and Sitting, demonstrated a different pathway to policy impact, so that the descriptive evidence they produced on “new” risk factors directly produced policymaker, community and media interest, which led to policy interventions. The assessment of the GenVape’s policy impact noted that “clear causal links between evidence and legislative change are documented, with the research leading to “policy | legislation introduced” (expert). For Sitting, policy impacts were in the form of professional and community guidelines. An expert commented that “the initial epidemiological work had a direct impact on international and Australian physical activity health guidelines, recognizing sitting’s role as a chronic disease risk factor. This has continued as a subset of physical activity policy and advice.” There were also practice impacts arising from the sitting workplace intervention studies, with widespread adoption of sit-stand desks across various workplaces and wide availability to the public. This practice impact was magnified through commercial interests and their development and marketing of sit-stand desks.

### Impact on individual researchers and organizational research capacity

The sitting case study was assessed as having a substantial effect on research capacity and impact, with GHS research and GenVape both receiving high impact ratings in this domain. The HSF case was rated as having moderate impact, which in part may reflect the smaller size of the project and team.

### Researchers

All four cases engaged PhD students; and this was reported as being valuable in terms of rigor, funding and achieving scientific outputs. For example, GenVape provided a platform for “ECRs [early-career researchers] and PhD students to gain experience and contribute to impactful research, as part of a team” (researcher). For GHS, one researcher explained how their doctoral study-built skills, “being able to do a PhD on it has exponentially increased my capacity, as an individual.” This research also engaged other researchers and biostatistician trainees who “utilized Get Healthy data and helping them to skill up, because the GHS is very complex and messy” (research). In the HSF research program, researchers were challenged by complex methodological issues, being “forced to do things which were quite difficult in terms of bias, statistics and analysis” (research), and it was noted that it “significantly enhanced the capacity of individual researchers involved, particularly in methodology and practical evaluation skills” (research). Importantly, it was reported that the applied nature of HSF research provided significant learning, enhancing researchers’ “understanding of policy processes and considerations,” fostering “real research policy dialogue going on the whole time” (researchers). Similarly, one Sitting researcher noted that this experience led them to “understand the importance of working with stakeholders and maintaining those relationships.”

### Impact on organizational research capacity

The results suggest that each case study bolstered the research capacity and public profile of collaborating organizations, including the PRC, Cancer Council NSW (an independent charity working in cancer research, prevention and information), and government agencies. GenVape “enhanced PRC’s profile due to high media, policy and public exposures” while bringing additional capacity at PRC, this collaboration with Cancer Council NSW helped address “university resource limitations by leveraging Cancer Council NSW’s infrastructure” (research) for communications and dissemination. The program enhanced Cancer Council’s “evidence-based advocacy capabilities” and provided a “stronger evidence base needed for more effective advocacy” (policy). For government agencies, the research “addressed critical data gaps” and “promoted their rapid response capacity” by sharing data “almost live.”

The GHS research “strengthened PRC’s track record in applied, scaled up research” (expert) and “[gave] some credibility to the translational focus and the applied focus that they (PRC) already had” (expert). A policy informant emphasized the institutional trust developed: “The PRC and their commitment to this space has shown the high level of impact and trust that NSW Health has in the evidence and review of the program. That trust supports program sustainability.”

The HSF research provided “another opportunity to showcase PRC’s work in undertaking real world evaluations” and “increased PRC’s profile across the Local Health Districts” (research). The program enhanced the Government’s “evaluation capacity and understanding of academia” (research) through collaborative methodology design and interpretation. The Sitting research “positioned PRC as a leading institution in this emerging field,” building on PRC’s expertise and experience in PA “and placing PRC at the forefront in Australia in investigating sitting as a risk factor.”

### Health system, population, and social impact

There was evidence of some specific impacts on these more distal outcomes and a view that there was further potential for such impacts. The GHS had an impact on the health system, in so far that it continues to provide a way of delivering government health services across NSW. As noted by a policy key informant it “supported sustained investment in the service and enabled the design of new variants” and “has become more connected to clinical services and part of a continuum of care, filling a service gap within NSW health services.” The HSF changed a very specific feature of the health system; although the extent to which changes have been maintained and significance at population level is unknown. For GenVape, the potential population health impacts were identified, but considered to be dependent on legislative enforcement. The initial epidemiological findings for Sitting rapidly generated widespread interest, leading to changes in guidance and workplace expectations, a shift in social norms and, in some cases, behaviors and practices: “walking meetings—everybody does it now” (policy) and “Trojan horse to change workplace culture, to think about end of trip facilities, to going to the gym in the middle of the day as opposed to before or after work” (policy).

### Conditions associated with degree of impact

The thematic analysis of interviews and surveys generated several key circumstances and conditions that consistently facilitated (and constrained) research and policy impact across case studies. Key enabling features noted above, include policy alignment of the research and collaboration with policy and practice stakeholders. These emergent enabling themes cover a continuum of interactive, complex and sustained connections, varying with context and time. The elastic quality of these key enabling themes is depicted in Fig. 3. Thus, while policy alignment in terms of content was an important enabler, each case’s connectedness with policy was further enhanced by the strategic timing of research and dissemination. For example, a GHS researcher noted “we would look at the information …to see almost in almost real time who was using the service and what promotions were working.” Exceptional alignment was also apparent in HSF research, addressing practical implementation factors including acceptability and productivity. Collaborative partnerships were described as most effective when there were exceptional degrees of mutual appreciation and respect. One GHS policy interviewee commented “It was unlike anything I’ve ever seen before; there was that mutual understanding, and I think respect on both parties.”

**Figure 3:**
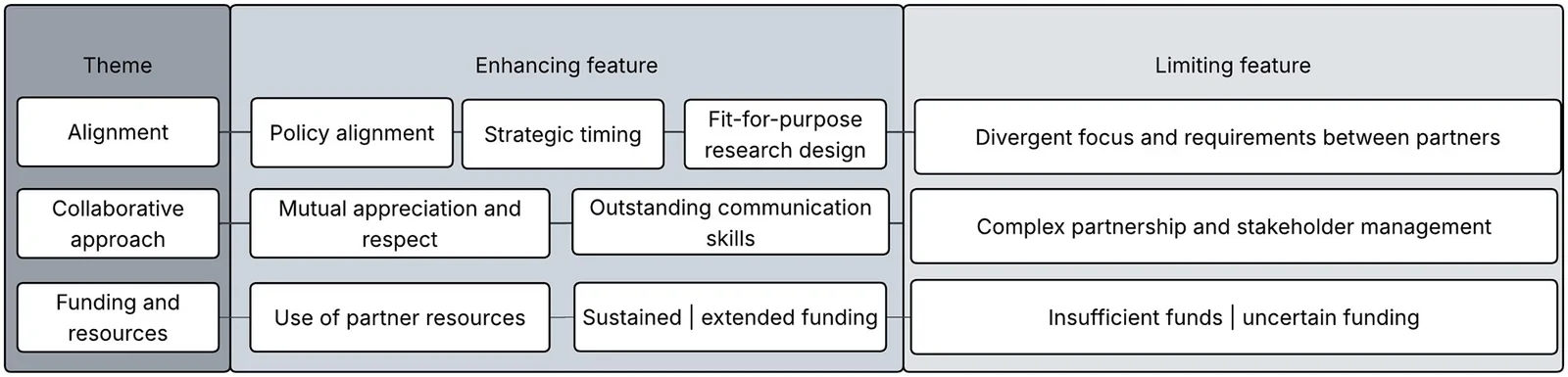
Enhancing and limiting features.

Each case study also encountered specific challenges or constraints in relation to these same contextual conditions. For example, all cases had base funding but nevertheless experienced funding uncertainties and limitations. Similarly, within the collaborations, informants reported the importance of managing relationships carefully, noting occasions when priorities and expectations diverged. For example, a HSF policy interviewee commented “we don’t necessarily need the level of academic rigor around some of that stuff to assert that we’ve done X. So it was challenging.” At the same time the researcher also considered the arrangement challenging, noting the limitations of “the short-term nature and specific contractual obligations.” Constraints were also related to the political nature of the policy context. For example, a GHS researcher noted “a new governance structure made it difficult for the evidence to be used appropriately.” A key challenge in policy-initiated cases derived from unequal power relations within the partnership; while investigator-initiated cases continually sought to maintain connection and traction with policy-makers.

## Discussion

### Impact and influence across domains

This study found consistent evidence that each case study contributed to building evidence in their topic area, provided opportunities for researchers to develop skills and experience in applied research, strengthened overall research capacity in the research organization, and importantly achieved policy or practice impacts. These impacts and influences were found across multiple domains. In particular, the inclusion in this study of knowledge development and research capacity impact domains threw light on a comprehensive set of related outcomes not always well documented [33, 34]. The case studies also identified subsequent social and health system changes and the potential to improve population health. Across the four research programs, policy, practice and research impacts tended to be immediate or mostly short-term; while longer-term impacts in relation to distal outcomes were more difficult to trace, and typically more diffuse and diluted.

This study advances a more nuanced appreciation of the spectrum spanning influence and impact, with the case studies illustrating the contributions of different types of evidence [35]. This reinforces the need to assess impacts relative to the purpose, scope and focus of the topic under investigation, as well as the research itself and stage of evidence building in that topic [34–36]. For example, some topics have broader significance, such as identifying a new, prevalent problem and may be viewed as having greater significance than refining or testing one component of a large-scale preventive strategy (as with Sitting and GenVape as compared to HSF evaluation). However, this does not diminish the real-world value of comprehensive evaluation research and emphasizes how varied types of research are essential for evidence-based policy [35, 37].

### Differences in trajectories

These research programs shared key features in how they were designed and conducted: alignment with global, national and/or state health priorities; funding; collaboration; and active dissemination and translation processes. Yet there were major variations in the types of research undertaken and evidence produced (as per Table 3), as well as timeframes and focus. In particular, their differing starting points, in terms of research type and whether they were initiated by government or researchers, was linked with distinct trajectories. The investigator-initiated programs had direct impacts in adding new topics or perspectives to national and international public health priorities, while policy-initiated research provided practice-based evidence on at-scale population interventions. Both trajectories influenced government decision-making.

Each case presents a balance of rigor and relevance, with peer-reviewed publications on highly applied, policy relevant research [38]. Context, topic, focus, duration, and research stage influenced publication extent; and citations indicated potential influences across locations and fields. Notably, the knowledge development scores in each case study were unrelated to publication volume. For example, the limited existing evidence base in the HSF topic made this research particularly valuable.

There was a clear contrast in the dissemination and translation approaches: for GenVape and Sitting, media was a significant channel; while government-funded cases prioritized direct and rapid feedback to the intervention agency (Government in these cases). This partly reflects media’s well-known interest in new problems and debates and lesser interest in practical solutions that lack “magic bullet” appeal [39, 40]. In all cases, translational actions and opportunities constituted best practice, as they were “fit for purpose,” shaped by the research characteristics and context [33, 41, 42]. This finding aligns with the literature on the wide range of translational strategies available and the need for researchers to make judgements about what is appropriate within the relevant implementation system and given context [43].

### Capacity building

PhD and early career research interviewees were clear that they learnt a great deal by being involved in highly applied research. Policy/practice interviewees also noted valuable learning through the collaboration. Interviewees and reviewers reported that each research program enhanced the capacity and reputation of the host research group, and particularly its capacity and skill to combine scientific rigor with real-world applications. The inclusion of impacts in these domains recognizes the importance of evidence-based public health capacity at both individual and organizational levels [44]. While these impacts are not easily quantified or corroborated, this reputation in turn contributes to collaboration and knowledge exchange between researchers and policy/practice agencies [45, 46].

### Enabling and hindering factors

Our findings on the factors that enabled impacts reflect previous research: policy alignment; sustained collaboration with applied partner agencies; a stable funding base; and active, targeted translational strategies [9, 12, 19, 27]. We found that translational efforts were embedded in collaborative partnerships, thus illustrating the importance of sustained relational arrangements within relevant implementation systems [33, 38, 41, 47]. That is, our findings show that traditional enabling factors can be enhanced; yet there can be times and circumstances where they operate as constraints. Overall, the case studies show the elasticity of enabling and hindering factors that applied research groups negotiate and manage. Effective translation is clearly not a checklist of activities or tasks but depends on sophisticated skills, sound experience, a collaborative organizational culture responsive to “windows of opportunity,” and capacity to foster sustained partnerships [33, 41, 47].

### Limitations and strengths

This study has several limitations. The case study sample was purposeful, and selection was guided by the need to find distinct, substantial research programs, acknowledging that this may result in selection bias. The four cases share some distinctive features. The degree of collaboration with policy/practice partners described is likely not typical of research programs. Each case study also had dedicated resources, which likely influenced numbers of publications, staffing, collaborations and dissemination efforts; however, this study could not examine funding levels. Some variation between cases corresponded to whether they were policy or investigator-initiated; while this variation supported comparison based on program origin, it was not sufficient to attribute differences in impact to any specific feature. While three case studies were completed before 2023, the GenVape case was ongoing at the time of writing, so its publication profile is expected to change over time. Additionally, the contemporary communication and social media context for GenVape differs from the earlier media context for the other cases. The interviews included policy and practice stakeholders where possible; however, staff turnover meant that some potential policy interviewees were unavailable. Furthermore, as key informants were mostly considering their work in hindsight, they may have given emphasis to positive aspects of collaboration and less emphasis on day-to-day experiences of frustration and complexity. The indicator tool used was new and designed to enable reviewers to make specific assessments within each domain. It is recognized that the impact ratings pertained to a specific point in time and do not provide a fixed account of impact; but relied on reviewers’ individual judgement at a given time and which could have contributed to scoring variation. In addition, interview and survey data were coded by a single researcher per case study rather than independently by multiple coders, which may have introduced coder-specific interpretation; this was partly mitigated by between researcher discussions and clarifications, a structured, deductive coding framework and by cross-case framework analysis, which checked patterns against multiple cases and data sources rather than relying on one coder’s reading of a single case.

This study drew on a variety and mix of data sources which yielded rich and diverse information [11, 13]; and the mixed-methods approach allowed limitations of individual methods to be balanced by others. Interview, surveys and document review provided indications of impact, while bibliometric indicators captured academic reach; however, neither fully evidenced impact on their own. Expert reviewer assessment, conducted independently of the authors and on the basis of these data sources, added structured, comparable judgement across cases, strengthening the validity of the overall impact assessment. Where expert ratings differed, review of their qualitative, free-text comments and rationale helped explain, validate, or qualify these ratings; with discrepancies typically reflecting differences in the timeframe or type of impact being considered, demonstrating how integration surfaced evidence that no single method would have captured alone.

## Conclusion

This study demonstrates a “helicopter” perspective seeking to trace the trajectory of selected research programs in a research- and teaching-intensive academic group [48]. It provides a comprehensive analysis that shows there is no single pathway to achieving impact and that research impacts vary according to the topic, the focus, and the stage of evidence-building. Different types of evidence support different impacts and influences; these operate along a spectrum and span not only policy capacity and impact but also knowledge development and research capacity.

This account specifically presents four cases where multi-study research programs made significant contributions to policy, practice, and research, highlighting the value of advanced, sophisticated translational approaches that are embedded in collaborations; and reinforces the role of an organizational culture that supports translation. These findings are likely most applicable to research groups with established policy networks, sustained relationships with partners, and dedicated translation capacity and resourcing, conditions that reflect the specific context of PRC described here. More generally this study illustrates how intensive case study methods can reveal the ways research become influential and impactful [49], adding new tools to the impact assessment repertoire.

## Data Availability

De-identified data from this study are not available in a public archive. De-identified data from this study will be made available (as allowable according to institutional IRB standards) by emailing the corresponding author.

## Artificial intelligence

Artificial intelligence was not used in the development of this manuscript.
